# Inhibiting autophagy overcomes docetaxel resistance in castration-resistant prostate cancer cells

**DOI:** 10.1007/s11255-018-1801-5

**Published:** 2018-02-19

**Authors:** Quan Wang, Wei-Yang He, Yi-Zhou Zeng, Arman Hossain, Xin Gou

**Affiliations:** 1 Department of Urology, The First Affiliated Hospital of Chongqing Medical University, Chongqing, 400016 China; 2 Central Laboratory, The First Affiliated Hospital of Chongqing Medical University, Chongqing, 400016 China

**Keywords:** Castration-resistant prostate cancer, Docetaxel resistance, Autophagy, Tea polyphenol

## Abstract

**Background:**

This study investigates the docetaxel-resistant mechanism and explores the effect of tea polyphenols (TP) on autophagy and its related mechanism in human castration-resistant prostate cancer (CRPC) cell lines PC3 and DU145.

**Methods:**

Immunofluorescence assay and annexin V-FITC/PI double staining flow cytometry were used to analyze the apoptosis and autophagy of PC3 and DU145 cells. The expression of autophagy-related proteins was detected by western bolt.

**Results:**

Docetaxel could induce autophagy and apoptosis, together with the expression increase in p-JNK, p-Bcl-2 and Beclin1. The level of autophagy was remarkably decreased, but apoptosis was increased after combining with TP. In addition, the expression of p-mTOR was increased after combining with TP.

**Conclusion:**

Docetaxel induces protective autophagy in CRPC cells by JNK pathway activation and then Bcl-2 phosphorylation and Beclin1 dissociation. TP activates mTOR pathway, which ultimately inhibits docetaxel-induced autophagy and improves therapeutic efficacy of docetaxel in CRPC cells.

## Introduction

Progression to castration-resistant prostate cancer (CRPC) is most common ending in patients with metastatic prostate cancer after a period of androgen deprivation therapy [1]. As an antimitotic chemotherapeutic, docetaxel is considered as a first-line therapy in CRPC [2], it only gives a moderate survival advantage as patients eventually acquire resistance [3]. Resistance to docetaxel is well characterized and attributes to numerous different mechanisms. Many of these mechanisms are related to abnormal molecular regulation, which involved in cell survival and death [4].

Macroautophagy is a cellular survival pathway and a stress response. It is responsible for the degradation of protein aggregates, long-lived proteins and damaged organelles to maintain cellular homeostasis [5]. The process begins when phagophores emerge and nucleate. Phagosomes elongate to form autophagosomes via two ubiquitination-like systems: the autophagy-related protein ATG12–ATG5–ATG16 system and the phosphatidylethanolamine-modified microtubule-associated protein light chain 3 (LC3-II) system. Autophagosomes fuse with lysosomes to form autolysosomes and then degrade their goods [6–9]. A lot of studies indicate that autophagy is stimulated under hypoxia and starvation via various tumor cell survival mechanisms, and inhibiting autophagy obviously increases tumor death and decreases tumor growth [10, 11]. Therefore, targeting autophagy to strengthen the therapeutic effects of anticancer drugs presents a new approach for cancer therapy.

Tea polyphenols (TP) are major biological active constituents of green tea, and the main component is catechins. Epigallocatechin-3-gallate (EGCG) is most affluent and may account for 50–75% of the catechins [12]. There are some studies showed that TP can be a good chemotherapy drug sensitizer [13, 14]. About the relationship between TP and autophagy, there is no authoritative conclusion. A study by Gu et al. [15] demonstrated that EGCG induced cells autophagy by the suppression of mTOR pathway. However, another research showed that EGCG stimulated autophagy and reduced cytoplasmic HMGB1 levels [16].

The Akt/mammalian target of rapamycin (mTOR) is regarded as the classical pathway for autophagy activation. Inhibiting the Akt/mTOR cascade obviously increases autophagy. Rapamycin (RAPA), a well-known mTOR inhibitor, is widely used as an autophagy inducer [17–19]. In addition, the mitogen-activated protein kinase (MAPK) family is also a significant mediator of autophagy. In this study, we proved that docetaxel induces cytoprotective autophagy in PC3 and DU145 cells by activation of C-Jun N-terminal kinase(JNK)and then induces the phosphorylation of Bcl-2, then disruption of Bcl-2/Beclin1 complex and finally release of Beclin1. TP can inhibit docetaxel-induced autophagy and increase apoptosis by the stimulation of mTOR pathway in CRPC cells.

## Materials and methods

### Materials

Antibodies for GAPDH (#2118), phospho-Bcl-2 (#2827), phosphor-mTOR (#2976), Beclin1 (#3495), phospho-JNK (#4668), JNK (#9252) and Alexa Fluor 488-conjugated IgG (#4408) were purchased from Cell Signaling Technology (Cell Signaling Technology, Danvers, MA, USA). Anti-PARP and p62 (610497) were purchased from BD Biosciences (New York, USA). Antibodies for LC3B (L7543) and chloroquine (CQ, 50-63-5) were purchased from Sigma-Aldrich (St Louis, MO, USA). All HRP-conjugated IgG secondary antibodies were purchased from Abgent (San Diego, CA, USA). The JNK inhibitor SP600125 (s1460) and docetaxel (s1148) were purchased from Selleck (Houston, TX, USA). The 3-methyladenine (3MA) (sc-205596) was purchased from Santa Cruz Biotechnology (Santa Cruz, CA, USA). TP (84650-60-2) was purchased from Yongye Biotechnology (Qingpu, Shanghai, China). Roswell Park Memorial Institute-1640 (RPMI-1640) and trypsin were purchased from HyClone (Logan, UT, USA). Fetal bovine serum (FBS) was purchased from Gibco (Thermo Fisher Scientific, MA, USA).

### Cell culture

The human prostate cancer cell lines PC3 and DU145 were kindly provided by Chongqing Key Laboratory of Molecular Oncology and Epigenetics (The First Affiliated Hospital of Chongqing Medical University, Chongqing, China). The cells were cultured in RPMI-1640 containing 10% FBS, 1 mmol/l-glutamine, 1 × 10^5^ units/L penicillin and 100 g/L streptomycin and maintained at 37 °C with 5% CO_2_ in a humidified atmosphere.

### Western blot analysis

To obtain the total protein lysates, treated cells were lysed in cold RIPA lysis buffer (Beyotime, Haimen, China) containing 1 mmol/L phenylmethanesulfonyl fluoride (PMSF; Beyotime, Haimen, China), 1% protease inhibitor cocktail and 1% phosphatase inhibitor cocktail (Biotool, Houston, TX, USA) and centrifuged at 12,000×*g* for 15 min at 4 °C to remove debris. Protein concentrations were estimated using the enhanced bicinchoninic acid (BCA) protein assay kit (Beyotime, Haimen, China), and the protein extracts were heat denatured in SDS-PAGE sample loading buffer (Beyotime, Haimen, China). Equal amounts of protein (40 µg/lane) from each sample were separated by 10–12% SDS–polyacrylamide gel electrophoresis (SDS-PAGE) using the criterion system at a constant voltage of 90 V. The proteins were subsequently transferred to polyvinylidene fluoride (PVDF) membranes (Merck Millipore, Darmstadt, Germany). After blocking with 5% nonfat dried milk for 2 h, the membrane was incubated with the primary antibodies overnight at 4 °C. Then, the immunoreactive bands were visualized by enhanced chemiluminescence using HRP-conjugated IgG secondary antibodies.

### Flow cytometric (FCM) analysis of apoptosis

After treatment, the cells were trypsinized, washed with PBS and suspended in 195 µL of annexin V-FITC binding buffer containing 5 µL annexin V-FITC and 10 µL propidium iodide (PI; Beyotime, Haimen, China). After incubation for 10–20 min at room temperature in the dark, the cells were subjected to a FCM assay. FCM was performed using a FACSCanto 6-color flow cytometer (BD Biosciences, San Jose, CA, USA).

### Immunofluorescence analysis

Cells were cultured on glass coverslips and fixed in 4% formaldehyde for 30 min at room temperature prior to detergent extraction with 0.1% Triton X-100 for 10 min at 25 °C. Coverslips were saturated with 2% bovine serum albumin (BSA) in phosphate-buffered saline (PBS) for 1 h at room temperature and processed for immunofluorescence with primary antibodies followed by Alexa Fluor 488-conjugated IgG (Cell Signaling Technology, Danvers, MA, USA). Nuclear morphology was analyzed with the fluorescent dye Hoechst 33342. Between all incubation steps, cells were washed three times for 3 min with 0.5% BSA in PBS. In brief, images were collected using a laser-scanning confocal microscope (Fluoview FV-1000; Olympus) using a 60 × Plan Apo/1.45 oil immersion objective and Fluoview software (FV10-ASW 1.6; Olympus). Images were subsequently analyzed for fluorescent intensity levels and co-localization of various stains by Image-Pro Plus 5.1 software (Media Cybernetics).

### Statistical analysis

All data are expressed as the mean ± SD. The SPSS 19.0 software package was used to perform all statistical analysis. Statistical comparisons were performed by one-way ANOVA. In all analysis, *P* < 0.05 was considered statistically significant.

## Results

### Docetaxel induces cell apoptosis and autophagy in PC3 and DU145 cells

**Fig. 1:**
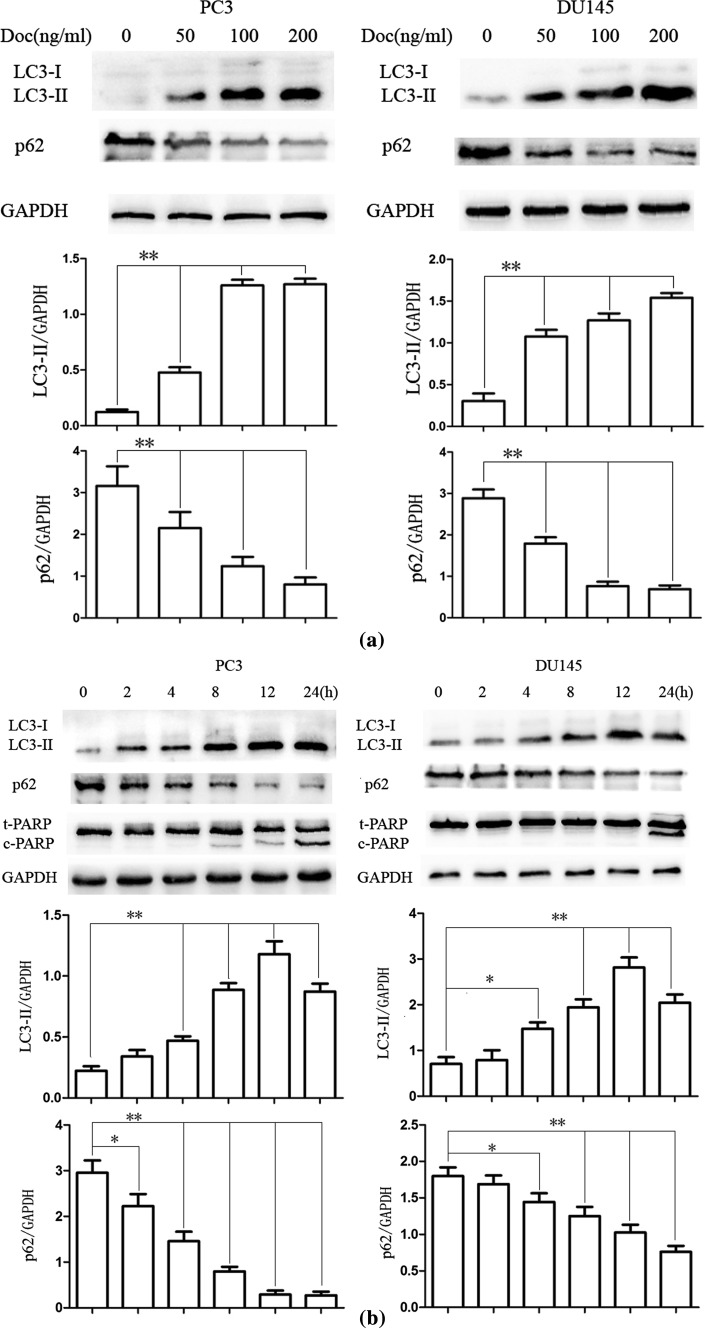

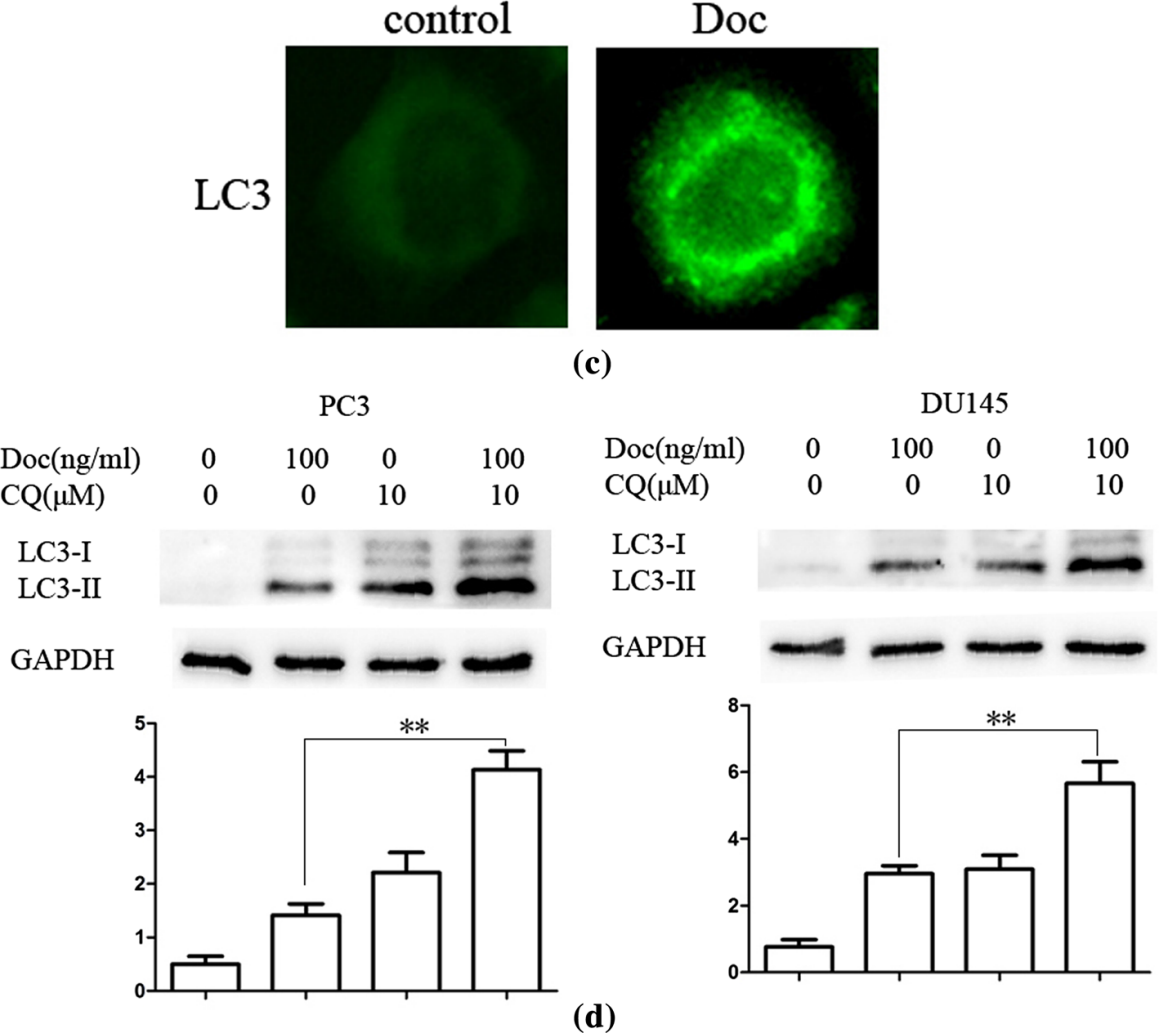
Docetaxel induces cell apoptosis and autophagy in PC3 and DU145 cells. **a** PC3 and DU145 cells were treated with various concentrations of Doc for 12 h, and cell extracts were analyzed to determine changes in protein expression by western blot analysis. **b** PC3 and DU145 cells were treated with Doc (100 ng/ml) for the indicated time. Western blot was used to detect protein expression. **c** PC3 cells were treated with Doc (100 ng/ml) for 12 h, and LC3 punctate formation was assayed by confocal microscopic analysis. Images are representative of 10 random fields. **d** PC3 and DU145 cells were cultured in Doc (100 ng/ml) for 12 h with or without CQ pretreatment (10 μM, 30 min). Cell extracts were analyzed for protein expression using western blot analysis. *P < 0.05, **P < 0.01

We initially investigated whether or not docetaxel could induce autophagy and the effects of docetaxel on apoptosis in PC3 and DU145 cells. Western blot assay was performed to examine the protein expression of LC3-II and cleaved (ADP-ribose) polymerase (c-PARP), LC3-II is essential for autophagy formation and mainly used as a protein marker of this phenomenon, and c-PARP is a biomarker of apoptosis [20, 21]. The results showed that docetaxel increased c-PARP expression for a 24-h docetaxel (100 ng/ml) treatment; LC3-II protein expression in CRPC cells increased in a concentration and time-dependent manner after docetaxel treatment compared with that in the control group, while the expression of p62 is opposite to LC3-II (Fig. 1a, b). In autophagy process, both of the conversion of LC3-I to LC3-II and the LC3-II degradation events can be seen sequentially [20], after 12 h, the degradation of LC3-II may be more than production; therefore, the expression of LC3-II at 24 h is less than that at 12 h. p62 was identified as one of the specific substrates that are degraded through the autophagy–lysosomal pathway [22–24]. This degradation is mediated by interaction with LC3, which is recruited to the phagophore membrane and remains associated with the completed autophagosome [25]. The degradation of p62 suggests that LC3-II increase not owed to suppression of LC3-II degradation, but attributed to the activation of autophagy. Immunofluorescence assay showed that there was an increase in endogenous LC3 punctate formation following 24-h docetaxel (100 ng/ml) treatment in PC3 cells (Fig. 1c). CRPC cells were pretreated with chloroquine (CQ, 10 μM, 30 min), which affects lysosomal acidification to inhibit autophagy [20]. Results suggested that docetaxel increased the protein expression of LC3-II when combined with CQ treatment (Fig. 1d), indicating that docetaxel induced autophagic flux.

### Docetaxel induces autophagy by JNK signaling pathway activation in PC3 and DU145 cells

**Fig. 2:**
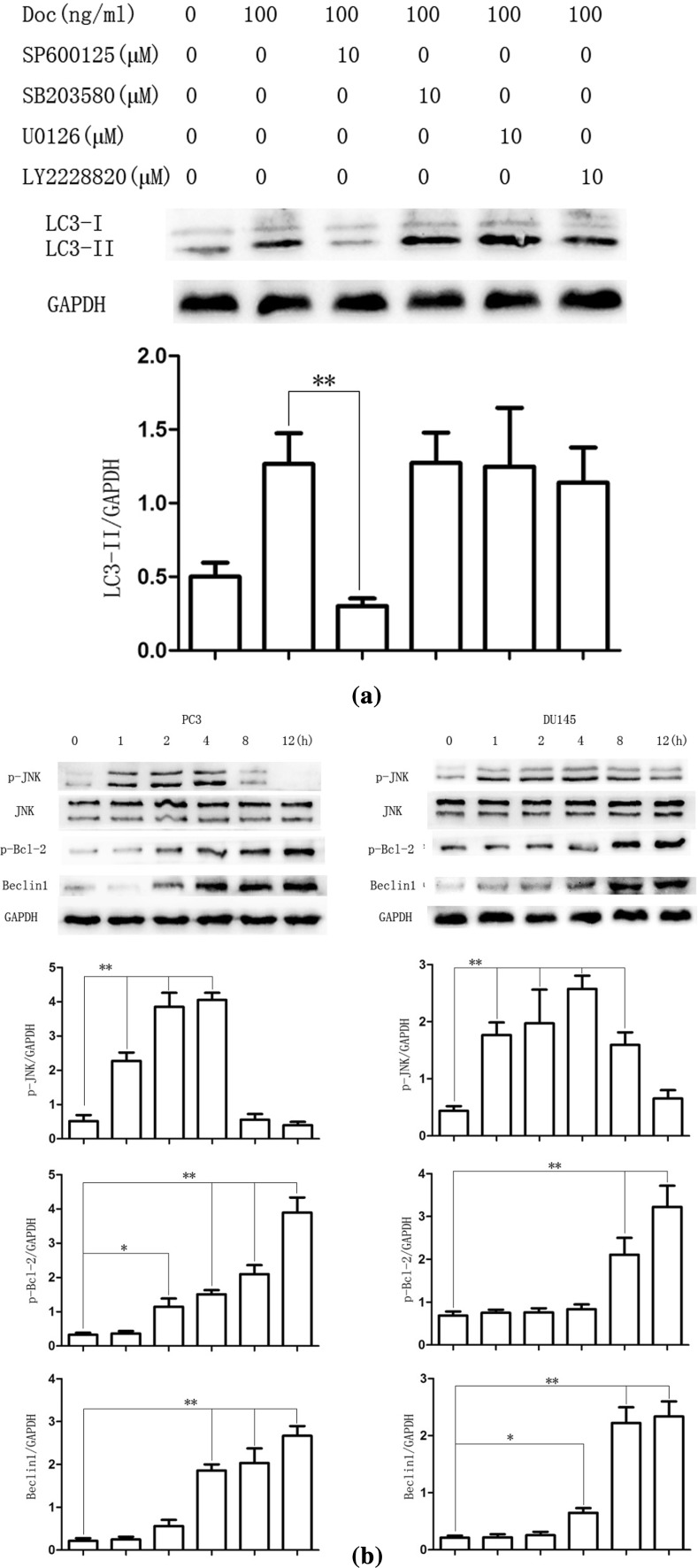

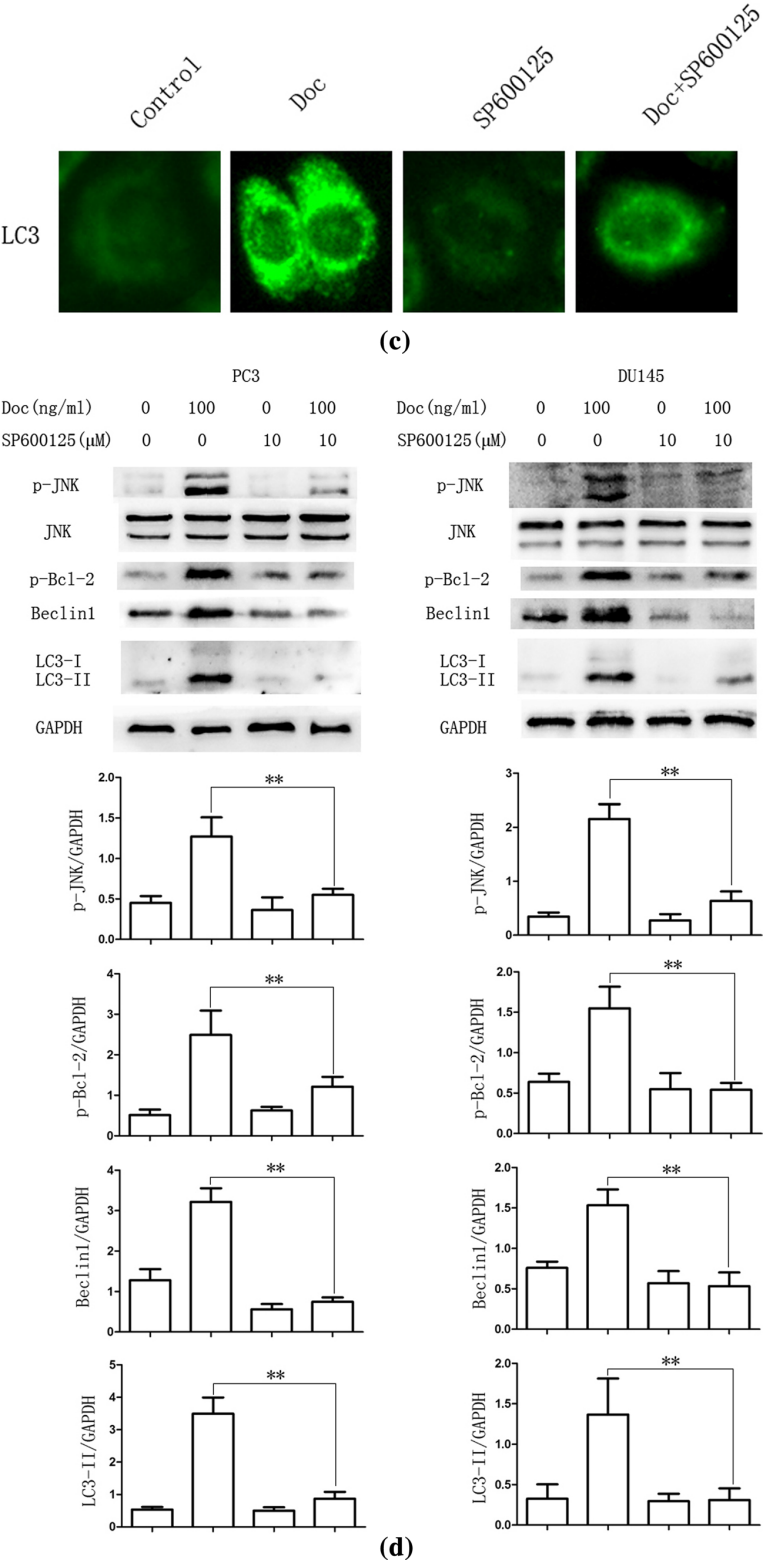
Docetaxel induces autophagy by JNK signaling pathway activation in PC3 and DU145 cells. **a** PC3 cells were cultured in Doc (100 ng/ml) for 12 h with different inhibitor (10 μM, 30 min). Cell extracts were analyzed for protein expression using western blot analysis. **b** PC3 and DU145 cells were treated with Doc (100 ng/ml) for the indicated time, western blot was used to detect protein expression. **c** PC3 cells were cultured in Doc (100 ng/ml) for 4 h with or without SP600125 pretreatment (10 μM, 30 min), LC3 punctate formation was assayed by confocal microscopic analysis. Images are representative of 10 random fields. **d** PC3 and DU145 cells were cultured in Doc (100 ng/ml) for 4 h with or without SP600125 pretreatment (10 μM, 30 min), western blot was used to detect protein expression. *P < 0.05, **P < 0.01

Activation of JNK was shown to induce the phosphorylation of Bcl-2, disruption of Bcl-2/Beclin1 complex and release of Beclin1 to induce autophagy [26]. As shown in Fig. 2b, Cells were treated with docetaxel (100 ng/ml) for 0, 2, 4, 8, 12 h, and western blot assay results showed that the expression of p-Bcl-2 and Beclin1 protein expression increased in a time-dependent manner after docetaxel treatment compared with that in the control group. However, the phosphorylated JNK was gradually increased until after 4 h of docetaxel treatment. Results suggested that JNK can be activated immediately when treated with certain compound and declined thereafter [27]. SP600125, a JNK pathway inhibitor [28], was used to examine whether docetaxel induced autophagy by JNK activation. As shown in Fig. 2c, docetaxel-activated JNK was decreased after pretreatment with SP600125 (10 μM, 30 min). The autophagy marker protein LC3-II increase was also reversed in PC3 and DU145 cells. In addition, compared with the group that cells treated with docetaxel alone, the expression of p-Bcl-2 and Beclin1 decreased in combined treatment. Immunofluorescence assay showed that LC3 punctate formation was particularly impair after combined with SP600125 in PC3 cells (Fig. 2d), suggesting that docetaxel induced autophagy via release of Beclin1 by JNK pathway activation.

### TP inhibits docetaxel-induced autophagy and promotes apoptosis in PC3 and DU145 cells

**Fig. 3:**
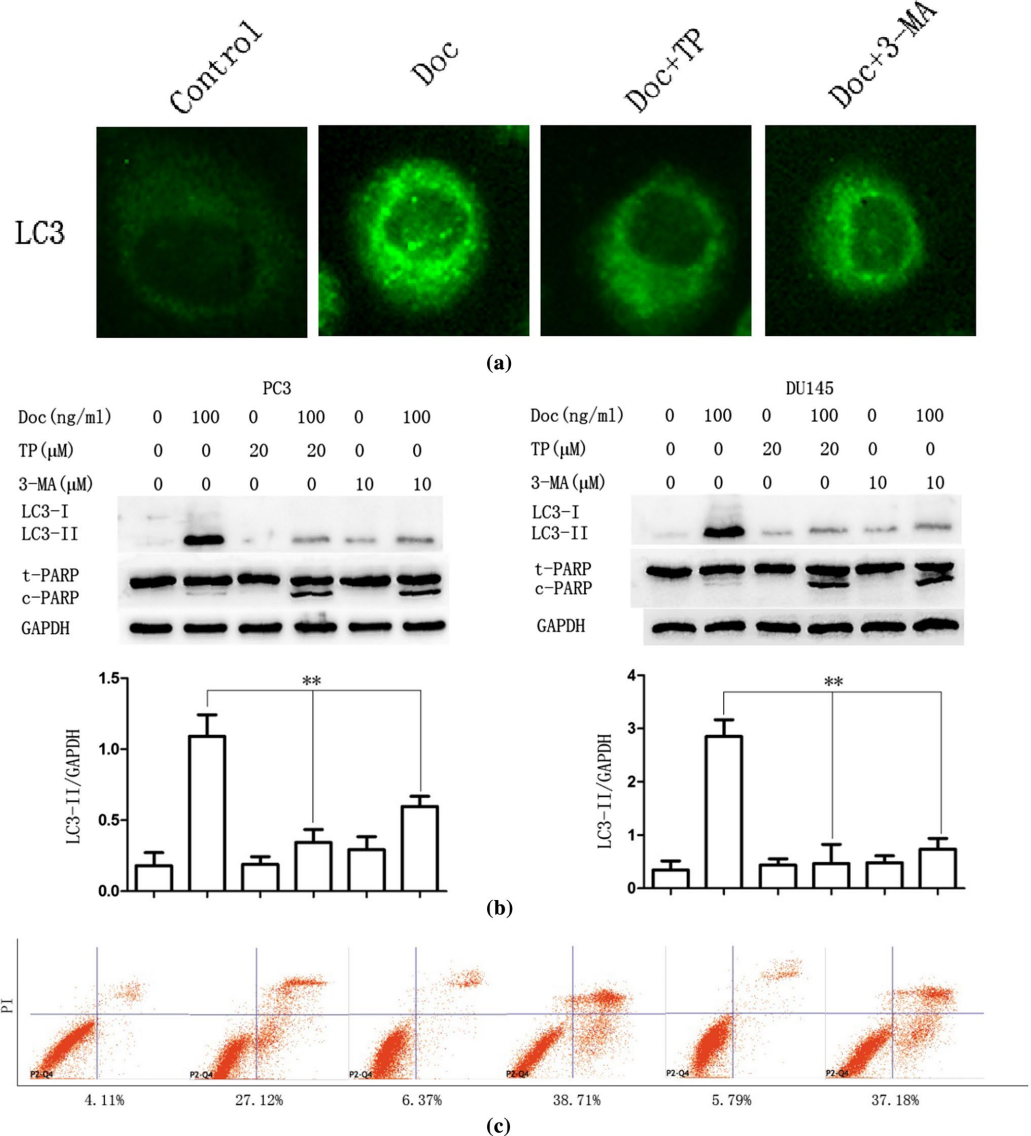
TP inhibits docetaxel-induced autophagy and promotes apoptosis in PC3 and DU145 cells. **a** PC3 cells were cultured in Doc (100 ng/ml) for 12 h with TP (20 μM, 30 min) or 3-MA (10 μM, 30 min) pretreatment, LC3 punctate formation was assayed by confocal microscopic analysis. Images are representative of 10 random fields. **b** PC3 and DU145 cells were cultured in Doc (100 ng/ml) for 24 h with TP (20 μM, 30 min) or 3-MA (10 μM, 30 min) pretreatment, cell extracts were analyzed for protein expression using western blot analysis. **c** PC3 cells were cultured in Doc (100 ng/ml) for 24 h with TP (20 μM, 30 min) or 3-MA (10 μM, 30 min) pretreatment, and cell apoptosis was measured by flow cytometry (FCM). The percentages of early and terminal stage apoptotic cells and necrotic cells were calculated. *P < 0.05, **P < 0.01

PC3 cells cultured in docetaxel (100 ng/ml) for 12 h with and without TP (20 μM, 30 min) pretreatment. Immunofluorescence assay results suggested that formation of LC3 punctate was impaired when docetaxel combined with TP (Fig. 3a). It demonstrated that TP could inhibit docetaxel-induced autophagy.

The effects of autophagy (pro-survival or pro-death) in cancer therapy were complex and inconclusive [29]. To further investigate the effects of TP combined with docetaxel on autophagy and apoptosis, PC3 and DU145 cells were pretreated with TP (20 μM, 30 min) before a 24-h docetaxel treatment. Western blot assay results showed that compared with that group which used docetaxel alone, the expression of LC3-II was markedly decreased and c-PARP protein expression increased that in docetaxel combined with TP group. For comparison, 3-methyladenine (3-MA), widely used as an autophagy inhibitor in vitro, was combined with docetaxel; we found similar result as TP combined with docetaxel (Fig. 3b). Flow cytometry was used to detect the apoptosis, as shown in Fig. 3c, more PC3 cells apoptosis occurred when combined with TP or 3-MA than treated with docetaxel alone. But there is no observably difference between TP and 3-MA.

### TP inhibits docetaxel-induced autophagy via mTOR signaling pathway activation in PC3 and DU145 cells

There is no acknowledged conclusion how TP inhibits autophagy and how to change autophagy at present. Although we found that TP could inhibit docetaxel-induced autophagy, the mechanism is not really clear. To researched mechanisms of docetaxel-induced autophagy inhibition of TP, the protein expression of high migration rate group protein B1 (HMGB1), JNK and mTOR activation was detected by western blot assay. HMGB1 is released by activated macrophages/monocytes [30]. A study by Li found that EGCG protects against lethal endotoxemia and sepsis by inhibition of HMGB1 [31]. The mTOR signaling pathway is known to regulation of autophagy, and mTOR inhibition results in the autophagy stimulation [15]. Because mTOR signaling plays an important role in a variety of protein expressions and functions, we also assumed mTOR signaling mediates the inhibiting effect of TP on docetaxel-induced autophagy in CRPC cells.

**Fig. 4:**
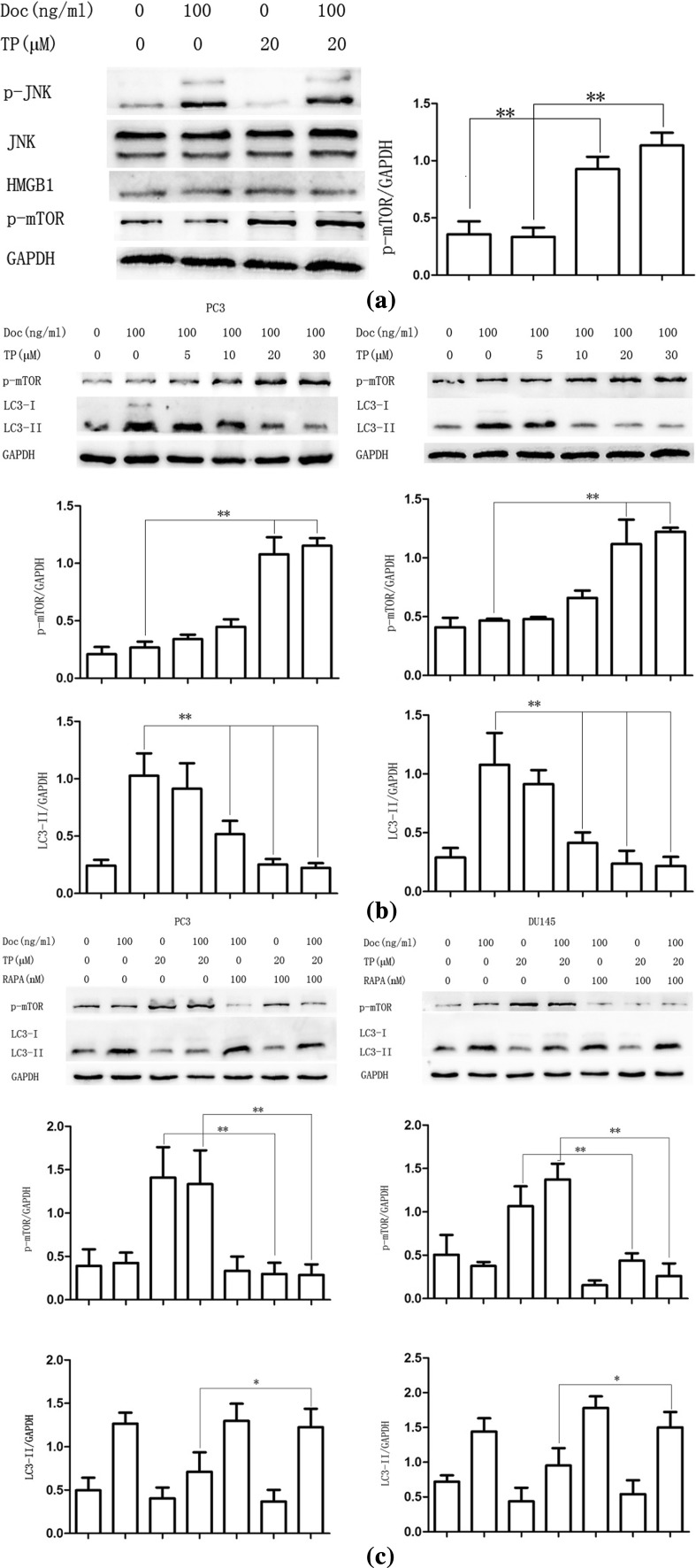
TP inhibits docetaxel-induced autophagy via mTOR signaling pathway activation in PC3 and DU145 cells. **a** PC3 cells were cultured in Doc (100 ng/ml) for 4 h with or without TP pretreatment (20 μM, 30 min), and western blot was used to detect protein expression. **b** PC3 and DU145 cells were cultured in Doc (100 ng/ml) for 4 h with a different concentration of TP pretreatment (30 min), and western blot was used to detect protein expression. **c** PC3 and DU145 cells were cultured in Doc (100 ng/ml) for 4 h with or without TP (20 μM, 30 min) or RAPA (100 nM, 30 min), and western blot was used to detect protein expression. *P < 0.05, **P < 0.01

We did not find the expression distinction of HMGB1 and JNK activation between combined treatment and docetaxel alone group; however, compared with control and only-docetaxel treatment group, the expression of p-mTOR is distinctly increased in that only-TP treatment and combined treatment group (Fig. 4a). The result indicated a fact that the effect of TP on CPRC cells is closely related to mTOR signaling pathway activation.

To find the relationship, PC3 and DU145 cells were cultured in docetaxel (100 ng/ml) for 12 h with a different concentration of TP pretreatment (30 min). Western blot assay results showed that p-mTOR protein expression in CRPC cells increased in a TP concentration-dependent manner compared with that in the only-docetaxel treatment group, while using docetaxel alone did not affect the expression of p-mTOR. In addition, LC3-II protein expression trend was opposite of p-mTOR (Fig. 4b). It indicated that mTOR activation may be involved in TP mediating the docetaxel-induced autophagy in PC3 and DU145 cells.

As a well-known mTOR inhibitor, RAPA is widely used as an autophagy inducer. In order to further understand whether TP mediates docetaxel-induced autophagy via mTOR signaling pathway or not, we used RAPA to inhibit mTOR pathway. As shown in Fig. 4c, TP could not enhance mTOR activation after mTOR pathway inhibited by RAPA, while the expression of LC3-II also did not decrease. It showed that inhibition of mTOR reversed the antagonism of TP on docetaxel-induced autophagy, and TP inhibits docetaxel-induced autophagy via mTOR activation in PC3 and DU145 cells.

## Discussion

Numerous studies have demonstrated that chemotherapy drugs can be induced autophagy, combined with autophagy inhibitor 3-MA, CQ, or silenced autophagy genes ATG5, ATG6, ATG7, ATG8 can significantly improve the therapeutic effect, reverse resistance of chemotherapy and ionizing radiation [32–35]. Cancer cells use autophagy to enhance their survival under harsh conditions of metabolic stress in the tumor microenvironment induced by chemotherapy and ionizing radiation. Docetaxel therapy has created clinical benefits for advanced prostate cancer; however, both acquired resistance and intrinsic resistance are universal outcomes. Various mechanisms of docetaxel-resistant exist in prostate cancer, such as ABC transporters [36, 37], glucocorticoid receptor (GR) [38], androgen receptor (AR) splicing [39, 40], epithelial plasticity [41, 42] and stem cells [43]. A better understanding of the mechanisms by which docetaxel resistance develops in prostate cancer can improve therapy strategies.

In our study, we found that docetaxel can change the expression of autophagy marker protein and induce autophagic flux in PC3 and DU145 cells, and it proves that docetaxel can induce CRPC cells autophagy. The MAPK family is an important mediator of autophagy, and some studies demonstrate that activation of extracellular signal regulated kinase (ERK) can induce autophagy [44–46]. We found that LC3-II expression decreased when PC3 cells had a treatment of docetaxel with pretreatment of SP600125 (10 μM, 30 min). And the expression of p-JNK had a time-independent increase for 0–4 h treatment; it suggested that docetaxel activates JNK pathway. At the same time, the Bcl-2 phosphorylation increased with the increase in docetaxel treatment time, and autophagy-related protein Beclin1 expression also gradually increased when prolonged the treatment of docetaxel. We put forward question as follows: whether or not docetaxel-induced JNK pathway activation mediated Bcl-2 phosphorylation, Bcl-2 dissociation from Beclin1 and then induced autophagy. To answer the question, SP600125 was used to inhibit JNK pathway, the results showed that p-JNK expression of drug combination group distinctly decreased, and it indicated that JNK pathway indeed inhibited. Nevertheless, the expression of LC3-II was decreased. It demonstrated that JNK pathway activation mediates docetaxel-induced autophagy in CRPC cells. After combined treatment with SP600125, the expression of p-Bcl-2 and Beclin1 no longer increased. These results proved that docetaxel induces CRPC cells autophagy by JNK pathway activation via Bcl-2 phosphorylation and Beclin1 dissociation.

The effects of autophagy on apoptosis travel both ways [47]. Previous studies indicated that activation of autophagy can limit a variety of drugs and stress-induced apoptosis [47–49]. This may be related to autophagy which can directly and specifically degrade unusual proteins and organelles which induce cells apoptosis [48]. On the other hand, excessive or deregulated autophagy can push the cells toward autophagic cell death. In our study, TP can inhibit docetaxel-induced autophagy in CRPC cells was proved; in addition, cells apoptosis was increased after autophagy inhibition. Autophagy early inhibitor 3-MA was used to contrast, we found the effect of TP on autophagy, and cells apoptosis had no evident difference. We came to the conclusion that docetaxel-induced autophagy is a protective mechanism to CRPC cells, it compromises therapeutic efficacy of docetaxel, and TP improves therapeutic efficacy of docetaxel and cells apoptosis by autophagy inhibition.

How TP affects cells autophagy after docetaxel treatment was a new question. Previous study reported that TP reduces cytoplasmic HMGB1 levels and then affects cells autophagy [16]. However, in our study, we did not find HMGB1 levels change in CRPC cells after TP treatment. We also detected p-JNK expression and found TP has no effect on JNK pathway. Nevertheless, compared with control and only-docetaxel treatment groups, the p-mTOR expression obviously increased in TP treatment and drug combination groups. Because the mTOR pathway plays a significant role in the number of protein expression and function adjustment, we conjectured that mTOR pathway mediates inhibition of TP on docetaxel-induced autophagy. TP was used to combine with docetaxel; the expression of p-mTOR increased while LC3-II expression decreased; when concentration of TP increased, the expression of p-mTOR had no obvious difference in that group used docetaxel alone. It demonstrated that TP can induce mTOR pathway activation, and this activation may be related to the inhibition of autophagy. Then, RAPA was used to inhibit mTOR pathway; we found that TP inhibiting docetaxel-induced autophagy in CRPC cells was reversed. The conclusion is that TP inhibits docetaxel-induced autophagy, improving therapeutic efficacy of docetaxel in CRPC cells by mTOR pathway activation. mTOR is a key regulator of the initiation phase of autophagy, and it regulates autophagy via multiple signals pathways [50]. It means that TP functions to reduce overall autophagy level to suppress survival capacity of prostate cancer cells.

In summary, docetaxel induces protective autophagy in CRPC cells by JNK pathway activation, then Bcl-2 phosphorylation and Beclin1 dissociation. Activation of mTOR signaling pathway by which TP inhibits docetaxel-induced autophagy improves therapeutic efficacy of docetaxel in CRPC cells. These findings suggest that TP should be evaluated in clinical trials as adjuvants with docetaxel in the treatment of docetaxel-resistant prostate cancer. At the same time, our study provides a potential strategy of CRPC therapy.
